# Utilizing customized CNN for brain tumor prediction with explainable AI

**DOI:** 10.1016/j.heliyon.2024.e38997

**Published:** 2024-10-09

**Authors:** Md Imran Nazir, Afsana Akter, Md Anwar Hussen Wadud, Md Ashraf Uddin

**Affiliations:** a Department of Computer Science & Engineering, Bangladesh University of Business & Technology, Dhaka, Bangladesh; b Department of Computer Science & Engineering, Sunamgonj Science and Technology University, Sunamganj, 3000, Bangladesh; c Department of Computer Science & Engineering, Jagannath University, Dhaka, Bangladesh

**Keywords:** Brain tumor, MRI, CNN, Explainable AI, SHAP, LIME, Grad-Cam, Diagonostic

## Abstract

Timely diagnosis of brain tumors using MRI and its potential impact on patient survival are critical issues addressed in this study. Traditional DL models often lack transparency, leading to skepticism among medical experts owing to their "black box" nature. This study addresses this gap by presenting an innovative approach for brain tumor detection. It utilizes a customized Convolutional Neural Network (CNN) model empowered by three advanced explainable artificial intelligence (XAI) techniques: Shapley Additive Explana-tions (SHAP), Local Interpretable Model-agnostic Explanations (LIME), and Gradient-weighted Class Activation Mapping (Grad-CAM). The study utilized the BR35H dataset, which includes 3060 brain MRI images encompassing both tumorous and non-tumorous cases. The proposed model achieved a remarkable training accuracy of 100 % and validation accuracy of 98.67 %. Precision, recall, and F1 score metrics demonstrated exceptional performance at 98.50 %, confirming the accuracy of the model in tumor detection. Detailed result analysis, including a confusion matrix, comparison with existing models, and generalizability tests on other datasets, establishes the superiority of the proposed approach and sets a new benchmark for accuracy. By integrating a customized CNN model with XAI techniques, this research enhances trust in AI-driven medical diagnostics and offers a promising pathway for early tumor detection and potentially life-saving interventions.

## Highlights

• First, this study introduced a customized CNN architecture specifically designed for brain tumor detection.
• Second, integrating SHAP, LIME, and Grad-CAM as XAI techniques enhances the model's clarity in the decision-making process.
• Moreover, the study validates the model across other datasets, demonstrating its robust performance and generalizability.
• This work opens the door to higher neuroimaging diagnostic accuracy by combining AI research and real-life clinical usage.

## Graphical abstract

**Figure:**
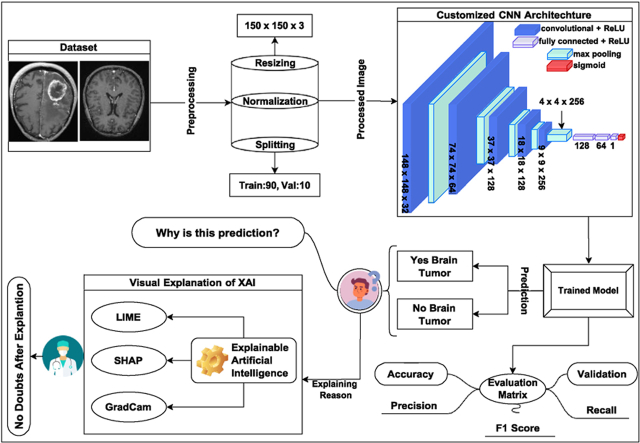

## 1 Introduction

The early detection of brain tumors is critical for saving lives and improv-ing patient outcomes. Brain disorders, including tumors caused by abnormal cell growth, present significant challenges in modern medicine and pose serious risks to individuals of all ages [1]. Brain tumors in the case of Low-Grade Gliomas (LGG) are often difficult to distinguish from normal brain tissue due to their slow growth rate [2]. On the other hand, High-Grade Gliomas (HGG) are a serious concern because they are the most aggressive type of tumor and tend to be malignant, usually seen as a mixed mass with surrounding edema [3,4]. In the field of medical diagnostics, magnetic resonance imaging (MRI), is a technology that has transformed the comprehension and visualization of the complex structures found within the human body. MRI serves as the gold standard and is the method of choice for the in vivo investigation of most brain diseases, such as different types of brain tumors [5]. MRI surpasses the conventional imaging methods. Its noninvasive nature allows doctors to examine internal structures without surgery. MRI is essential in modern healthcare, as it can accurately detect tiny changes in organs and pinpoint brain problems because accurate detection of brain tumors from MRI images is vital for effective treatment planning and improving patient outcomes. Traditional methods, which rely on manual interpretation by radiologists, can be labor-intensive and subject to human error.

However, the integration of artificial intelligence (AI) in medical imaging, particularly when using CNN, introduces the challenge of model interpretabil-ity. DL has significantly advanced medical imaging and offers precise disease classification and diagnosis [6]. However, the black-box nature of CNNs raises concerns regarding understanding their decision-making processes. To address this, XAI techniques, such as SHAP, LIME, and Grad-CAM, are employed to clarify the workings of these models. SHAP provides sophisticated knowledge of feature importance by valuing the contribution of each feature to the model's output [7]. It is based on the cooperative game theory. Meanwhile,

LIME provides insights into particular cases by producing locally faithful explanations through data modification and prediction change observation. Grad-CAM is a visualization technique used to understand the decision-making process of a neural network, particularly in image-classification tasks. It generates a heatmap that highlights the regions of an input image that contribute the most to a specific class prediction. By understanding the complicated interactions between the input feature choices and model output, these approaches can serve as interpreters.

However, there are obstacles to the use of transparent AI in medical imaging. Complete interpretability is a difficult goal to accomplish because of the complexity of CNNs and millions of parameters. Turning the vast amount of data these models contain into insights that are accessible to humans is the difficult part. Oversimplification may result in the loss of important details in the medical data; thus, it is important to maintain a careful balance between model complexity and interpretability. To address this gap, the present work focuses on the technical optimization of MRI images and analysis to identify brain tumors [8]. Research on improving XAI techniques is necessary because of the inherent trade-off between transparency and accuracy. This is to ensure that the techniques not only explain model predictions but also capture the complex intricacies present in medical images. Therefore, it plays a significant role in the improvement of treatment and human health [9].

The motivation for researching and developing an MRI tumor detection model was the possibility of saving lives by quickly and accurately identifying brain tumors. The primary idea was to understand that there could be meaningful benefits to patients if XAI could be used to improve the accuracy of predictions. XAI aims to overcome the problem of interpreting the decision-making of an ML algorithm when predicting the survival rate of patients with brain tumors based on MRI scans [10]. The dedication to this initiative was inspired by the possibility of helping to improve patient outcomes by offering a tool that enables more successful early detection and treatment.

MRI has become the preferred approach for brain tumor evaluation [11]. In this study, the model was trained using MRI datasets for tumor prediction to precisely identify brain tumors using a customized CNN model. Several convolutional layers were used in the model design to extract features, and fully connected layers were used for classification. XAI techniques have also

been applied to better understand the inner workings of MRI tumor detection models. In particular, utilized LIME, SHAP, and Grad-Cam to explore the CNN decision-making procedures. Confidence in the model's predictions was increased by these techniques, which also gave us a clear understanding of the elements impacting the model's judgments and a transparent glimpse into how it interprets various MRI image features.

This study makes significant advancements in brain tumor detection from MRI images by introducing a highly accurate, explainable, and generalizable deep-learning model. These are the key contributions of the present study.

• First, this study introduced a customized CNN architecture specifically designed for brain tumor detection. This model achieved an exceptional accuracy of 100 % and a high validation rate of 98.67 %, demonstrating its effectiveness in accurately identifying tumors on MRI scans.
• Second, the integration of SHAP, LIME, and Grad-CAM as XAI tech-niques enhances the transparency of the decision-making process of the model. This transparency is crucial for gaining insight into how the model arrives at its predictions, which is vital for medical professionals seeking to understand and trust AI-driven diagnostic tools.
• Moreover, the study validates the model across other datasets, demon-strating its robust performance and generalizability. This validation process ensured that the model maintained consistent accuracy and reliability across different imaging conditions and patient demographics, making it applicable in diverse clinical settings.
• By bridging the gap between AI research and practical clinical appli-cations, this study sets the way for improving diagnostic accuracy in neuroimaging. Early detection facilitated by the model's high accuracy could potentially lead to timely interventions and personalized treat-ment strategies, thereby improving patient outcomes and healthcare efficiency.

The rest of the paper is organized as follows: Section 2 highlights the Related Work. Section 3 analyzes the methodology. Section 4 presents the analysis and evaluation of the results. Finally, Section 5 concludes this paper.

## 2 Related Work

Deep learning (DL) has made significant progress in the detection and classification of brain tumors. This section reviews different studies utilizing diverse models and XAI techniques, analyzes their performance, and identifies limitations in their work.

Ullah et al. [12] presented a comprehensive approach to brain tumor classifica-tion using a hybrid methodology, achieving accuracy rates, with the combined Gabor filter and ResNet50 features yielding the highest performance, an accuracy of 95.73 %, precision of 95.90 %, and F1 score of 95.72 %. This study fills a crucial gap in the literature by addressing the challenges of early tumor detection using DL techniques. Future research could focus on improving real-time efficiency and validating the methodology across diverse datasets. Haque et al. [13] introduce NeuroNet19, attaining 99.3 % accuracy and 99.2 % precision/recall in brain tumor classification, using pre-trained models like ResNet50, VGG16, and VGG19. They highlighted the role of iPPM integration in improving feature extraction to address MRI scan variation. Ad-ditionally, the study underscores NeuroNet19's transparency through LIME, elucidating the critical image regions for prediction. Future directions include addressing binary-class limitations, enhancing the applicability of CT scans, and prioritizing dataset expansion.

Keles et al. [14] demonstrate the effectiveness of gradient-based saliency maps in brain tumor classification, emphasizing the importance of tumor presence and help in model decisions. They emphasize preprocessing steps, such as bone extraction, to boost accuracy, particularly for tumors with complex shapes. Future research on disease classification could employ similar XAI methods to enhance the model transparency and accuracy.

Bibi et al. [15] present a significant advancement in DL for neuroimaging, specifically in the classification of brain diseases using MRI scans. Their use of the DenseNet121 architecture achieved an impressive accuracy of 99 %, surpassing previous studies and demonstrating the potential of the model for clinical application. The integration of XAI techniques such as Grad-CAM enhances the interpretability of the model's decisions. The study acknowl-edges limitations, such as data dependence and potential variability in clinical settings.

Kumar et al. [16] introduced a Subtractive Spatial Lightweight (SSLW) CNN for brain tumor classification, achieving an accuracy of 80.15 % with reduced computational time compared to existing models. The study highlights the importance of XAI methods such as Class Activation Mapping (CAM) in enhancing model interpretability and trustworthiness, with CAM showing compatibility with human decisions and achieving a visual match rate of 86%–95 %. Despite the positive outcomes, further exploration of XAI methods and model formation is needed to enhance accuracy.

Sathyarajasekaran et al. [17] evaluate the efficacy of XAI algorithms in brain tumor segmentation, employing the Modality-Specific Feature Importance (MSFI) metric alongside Mutual Information (MI) and Understandability. While Guided GradCAM showed significant outperformance (p < 0.01), the overall MSFI scores remained relatively low, suggesting opportunities for improvement in XAI interpretability. This study highlights the role of MSFI in segmentation and the importance of human interpretability in medical imaging, offering valuable directions for improving XAI algorithms, building trust, and guiding future research.

Akter et al. [18] conducted an extensive investigation into brain tumor classi-fication and achieved notable accuracies ranging from 94.74 % to 98.8 %. Their study highlighted the effectiveness of dataset augmentation and transfer learn-ing models, underscoring their role in improving classification performance. In addition, the model attained an accuracy of 98.8 %, demonstrating the efficacy of their DL approach. However, this study suggests further refinement of the segmentation algorithms for enhanced accuracy.

Ranjbarzadeh et al. [19] presented an optimized CNN [20,21] model with 97.41 % precision, 95.78 % Recall, and a Dice score of 97.04 % for brain tumor segmentation on the BRATS 2018 dataset. Their focus on feature selec-tion and preprocessing improves the model performance, surpassing existing methods. Despite challenges such as fuzzy borders, their approach enhances the dataset balance and segmentation outcomes. Further research is needed to improve border detection and optimize feature selection to improve the accuracy.

Nhlapho et al. [22] provided a critical assessment of DL models for brain tumor classification, highlighting DenseNet121 and EfficientNetB0 as top performers with accuracy values of 97–98 % and significantly fewer parameters than VGG16 and VGG19. Grad-Cam and Grad-CAM++ were instrumen-tal in enhancing model interpretability, although challenges persisted with Integrated Gradient and Saliency Mapping. This study emphasizes the im-portance of balancing model complexity and accuracy.

Srinivas et al. [23] critically evaluated the performance of pre-trained CNN models VGG-16, Inception-v3, and ResNet50—on a dataset comprising 253 brain MRI images for tumor classification. The findings highlight VGG-16's superior accuracy (96 %) compared to Inception-v3 (78 %) and ResNet50 (95 %), although issues of overfitting were observed in the latter models.

Nassar et al. [24] present a robust hybrid technique for brain tumor classifi-cation, achieving 99.31 % accuracy through majority voting among CNN [25] models, with NASNet-Mobile leading at 98.3 %. This study underscores the efficacy of combining models for enhanced accuracy, highlighting the potential for future medical image analysis research. Acknowledging dataset limita-tions, especially in meningioma samples, suggests the need for broader data collection. This technique shows promise for aiding radiologists and reducing misclassification rates.

Gaur et al. [26] introduce an explanation-driven DL model achieving 94.64 % accuracy in brain tumor prediction from MRI images (meningioma, glioma, and pituitary), addressing interpretability concerns. They preprocessed MRI images, resized them to 150 × 150 × 3 pixels, and introduced Gaussian noise to improve DL learning. Their approach incorporated a dual-input CNN architecture and integrated LIME and SHAP to enhance transparency.

Zeineldin et al. [27] introduced TransXAI, a CNN-Transformer model for glioma segmentation in brain MRI. It enhances interpretability using Grad- CAM and aligns with clinical practice. Clinical feedback emphasizes trust and transparency. Despite these competitive results, dataset variability and MRI modality selection require careful consideration.

Sasmitha et al. [28,29] present two studies focusing on brain tumor diagnosis using MRI and whole slide imaging (WSI). The first study developed a Tumor Analyzer, achieving 86 % accuracy with DenseNet and ResNet models for MRI, enhancing interpretability with Grad-CAM for MRI, and a gated attention network for WSI. The second study integrates U-Net for segmentation and DenseNet for classification, achieving Dice coefficients of 0.7787 (ET), 0.8849 (WT), and 0.8041 (TC) for segmentation, and balanced accuracy, F1-score, and Cohen's kappa coefficient of 0.899, 0.857, and 0.733 for classification. Limitations include the computational expense of labeling MRI datasets and challenges in resizing Grad-CAM outputs from the deeper layers. Future work will aim to expand the input modalities and transition to desktop applications. Ullah et al. [30] introduce DeepEBTDNet, achieving an accuracy of 98.96 % in validation and 94 % in testing. Cross-dataset validation achieved an accuracy of 95.83 % for unseen samples. Incorporating LIME enhances interpretability and addresses blackbox concerns. Further research is needed to assess the performance across diverse datasets and mitigate LIME's computational costs.

### 2.1 Limitation of previous studies

Previous studies in the field of brain tumor detection using MRI have made significant strides; however, they have limitations. These limitations are listed in Table 1.

**Table 1:** Limitations of the related work.

Dataset Type	Year	Methods	Accuracy	Limitation	Ref.
Brain MRI images	2024	CNN	94.60 %	1. Computational time increases due to feature fusion	[31]
				2. Accuracy can be improved	
				3. No use of XAI	
Brain MRI images	2024	ARM-Net	96.64 %	1. Interpretability of ARM-Net's decisions not validated.	[32]
				2. Accuracy can be improved	
Brain MRI images	2023	Modified ResNet50 with HOG	88 %	1. Incomplete details on data augmentation strategies.	[33]
				2. Accuracy can be improved 3. No use of XAI	
Brain MRI images	2023	CNN with GNN	95.01 %	1. May not generalize to other datasets.	[34]
				2. Accuracy can be improved	
				3. No use of XAI	
Brain MRI images	2023	TECNN	96.75 %	1. High model complexity may hinder practical deployment.	[35]
				2. Accuracy can be improved	
				3. No use of XAI	
Brain MRI images	2022	EfcientNet-B0	98.97	1. Insufficient exploration of hyperparameter sensitivity	[36]
				2. Accuracy can be improved	
				3. No use of XAI	
Brain MRI images	2020	Hybrid & Single ResNet50	97.02 % 92.53 %	1. Limited discussion on the computational efficiency.	[37]
				2. Accuracy can be improved	
				3. No use of XAI	
Brain MRI images	2020	VGG16	88 %	1. Manual MRI annotation biases affect classification accuracy.	[38]
				2. Accuracy can be improved	
				3. No use of XAI	

After comprehensive research on previous studies, several limitations have been identified in brain tumor detection using brain MRI images, including low accuracy. Additionally, there is a notable absence of XAI techniques to enhance transparency and trust in the model's predictions. Recognizing these gaps, the proposed study aims to address these limitations and achieve higher accuracy while incorporating XAI methods for better interpretability.

## 3 Methodology

**Fig. 1:**
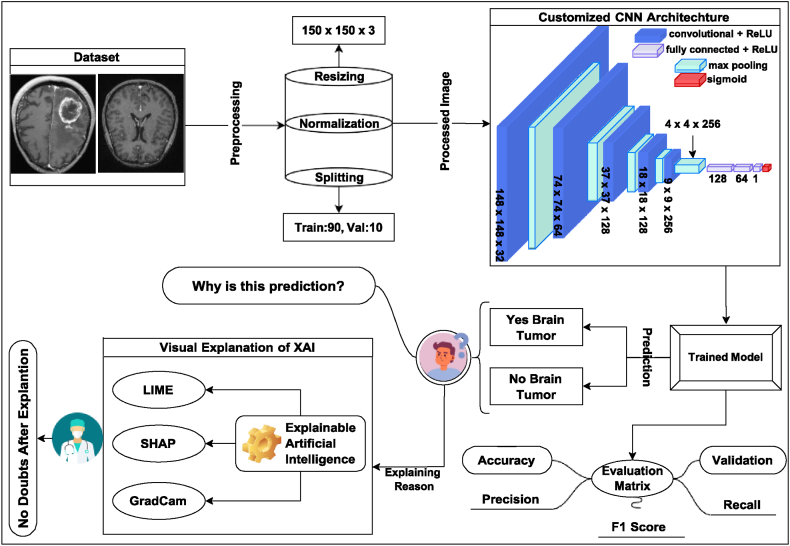
Proposed system architecture.

This methodology section presents the working procedure which is il-lustrated in Fig. 1. In addition, applied data splitting and different XAI techniques, such as SHAP, LIME, and GRAD-CAM. Finally, include the proposed model of this research.

**Fig. 2:**
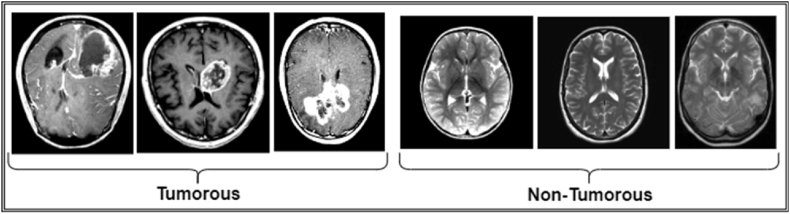
Sample images from the BR35H dataset: three images depicting tumorous tissue and three images depicting non-tumorous tissue.

The raw dataset ’BR35H’ [39] was collected from the open-source plat-form Kaggle, which consists of three main folders: "yes," "no," and "pred," containing a total of 3060 Brain MRI Images. Specifically, the "yes" folder contains 1500 brain MRI images showing tumors, while the "no" folder in-cludes 1500 images depicting non-tumorous brains. Visual samples of the dataset are shown in Fig. 2. This dataset provides a balanced representation of both tumorous and non-tumorous cases, facilitating the effective training and testing of machine learning models for brain tumor detection. This dataset is particularly suitable for the research because it provides a diverse range of images that enhance the robustness and generalizability of the model. In addition, the BR35H dataset is publicly available and has been used in various previous studies, allowing for a reliable benchmark to compare the results with existing models.

### 3.1 Data preprocessing

The dataset pre-processing involved several key steps. Initially, the images were labeled, followed by resizing to ensure consistent dimensions. Subse-quently, normalization was applied, and the dataset was split for further analysis.

Data Preprocessing: The preprocessing phase of the BR35H dataset is a critical preparatory step for optimizing the performance of deep-learning mod-els. Initially, the unlabeled nature of the dataset was addressed by assigning labels to each image. Tumor images were labeled 1, whereas non-tumor images were labeled 0. This labeling process laid the groundwork for supervised learning, enabling the models to accurately differentiate between tumorous and non-tumorous brain MRI images.

Resizing Images: Changing an image's dimensions without compromising its essential information is known as resizing. All images were resized to standardized dimensions of 150 × 150 pixels. This resizing step is crucial for maintaining consistency in the input size across the dataset, facilitating seamless integration into the DL pipeline.

Normalization: Normalization is a crucial step in preprocessing that re-turns the image pixel values. The applied normalization and pixel values were normalized to between 0 and 1. This is achieved by dividing each pixel value by the maximum possible pixel value, which is 255, in the context of the RGB color scale. By maintaining all input features on the same scale, it provides a more continuous completion of the optimized approach.

Data Splitting: Owing to the limited size of the dataset (3060 images), careful consideration was given to the data splitting strategy. The dataset was partitioned into training and validation sets, with 90 % (2700 images) allocated for training and 10 % (300 images) allocated for validation. This partitioning scheme ensures that the models are trained on a sufficiently large portion of the data while retaining a separate subset for unbiased validation purposes. The details of the dataset split are provided in Table 2, which shows the distribution of images into training, testing, and validation sets.

**Table 2:** Split of the BR35H dataset into the training, validation, and test sets. The training set contains 90 % of the images, the validation set contains 10 %, and the test set given in the prediction folder contains 60 images.

Dataset Details	Training	Validation	Testing	Total
Tumorous	1350	150	30	1530
Non-Tumorous	1350	150	30	1530
Total	2700	300	60	3060

### 3.2 Explainable AI techniques

Different XAI techniques were applied in this model to accurately un-derstand the internal working procedures of the model and how it makes decisions using SHAP, LIME, and Grad-CAM.

SHAP: SHAP values are used to determine the complex decision-making process of the neural network. They are a computational approximation of Shapley values, a method for assigning payouts to players in a cooperative game, or, in this case, contribution values to features in a prediction task. Training examples that comprise a context set provide a benchmark for the feature contributions. The trained model and backdrop data were used to ini-tialize the Deep Explainer, thereby making it possible to calculate the SHAP values for certain test images. These numbers show how individual pixels affect the predictions, which makes it easier to understand which features have a significant impact on the model's output. The outcome images help to create a more transparent and understandable neural system by providing detailed knowledge of the model's decision boundaries.

LIME: This method uses a model-agnostic methodology to explain the behavior of local predictions. The local model behavior is approximated around a set of test images using modified samples, which are defined as a prediction function and initialized as a LIME explainer. The creation of an interpretable surrogate model makes it easier to pinpoint important features, and the resulting visualizations draw attention to the precise areas that influence the model predictions. LIME improves the transparency of individual predictions by emphasizing local interpretability and providing important insights into the neural network's decision-making at the instance level.

Grad-CAM: In the field of image classification, in particular, the Grad- CAM technique is a useful method for improving the understanding of DL models. Grad-CAM generates informative heat maps that emphasize areas that are crucial to the decision-making procedure of the model. Without specifically identifying the framework, the code utilizes a trained brain-tumor detection model. After loading an image, preparing it, and running the Grad- CAM method, the last convolutional layer is used to extract the features. Subsequently, the original image is layered with the generated heatmap, which shows the key regions that influence the predictions of the modifications.

### 3.3 Proposed model

**Fig. 3:**
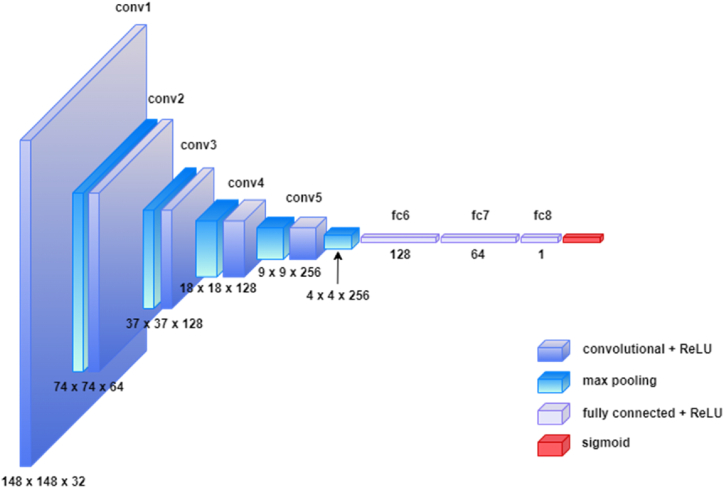
Proposed customized CNN model.

This study presents an innovative methodology for brain tumor detection employing the customized CNN model shown in Fig. 3, enhanced by the integration of three advanced XAI techniques: SHAP, LIME, and Grad-CAM.

This research developed a customized CNN specifically designed to identify brain tumors. Customization involves designing the architecture to enhance the feature extraction and classification accuracy specific to medical imaging challenges. The model begins with an initial layer that has 32 filters and progresses through layers with 64, 128, and 256 filters using a 3 × 3 kernel size for each. These layers are designed to incrementally capture more complex features in brain imaging data. To keep the model efficient and focused on important features, including activation functions called ReLUs (Rectified Linear Units) and max pooling steps that help reduce the size of the data passing through the network. After processing through these layers, the data are flattened and passed through a series of dense layers with 128 neurons and 64 neurons to finalize the decision-making process of the model. In addition,

a dropout layer reduces the chance of overfitting, which means that the model is more reliable when analyzing new, unseen brain images. The final decision of whether a tumor is present is made using a sigmoid function, which is well suited for binary classification tasks.

To address the black-box nature of CNNs, this study enriches the model using three XAI techniques: SHAP, LIME, and Grad-CAM. These tools help us peel back the layers of the CNN's decision-making process, making it easier to understand why it predicts the presence or absence of a tumor. This transparency is crucial, especially in the medical field, where understanding the ‘why’ behind a diagnosis is as important as the diagnosis itself. This approach aims to achieve high diagnostic accuracy with an open window in the model's thought process, making it both powerful and accessible to users, regardless of their expertise in AI. Moreover, the model consists of 1,511,233 parameters, which optimizes the performance while efficiently utilizing ap-proximately 5.76 MB of memory. During training, each epoch was completed in a swift 4–5 s on average, underscoring its computational efficiency and suitability for demanding tasks. This model supports deployment in CPU-based standalone applications, GPU-accelerated web deployments, and mobile applications that require high-performance and real-time inference.

Algorithm 1 Brain Tumor Detection using CNN and XAI.

**Figure:**
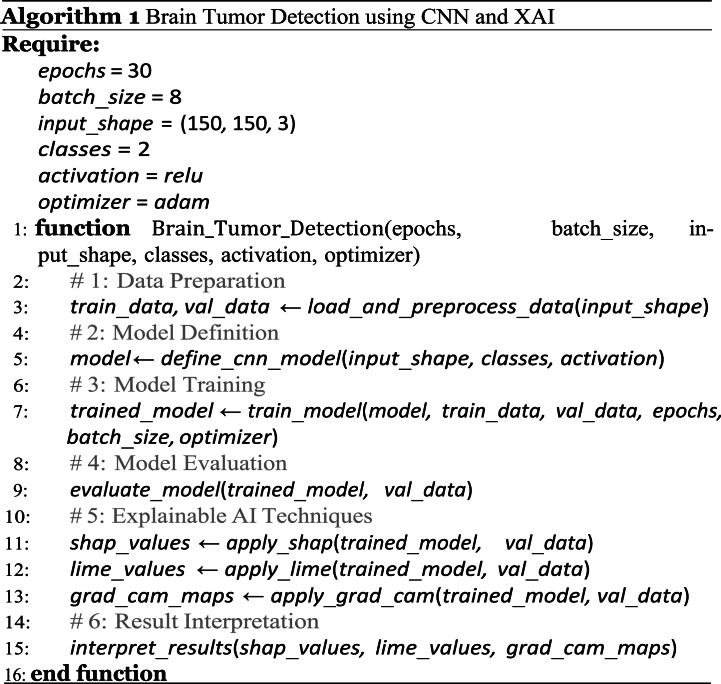

To better understand the work procedure of this study, Algorithm 1 is included, which outlines the entire process, from dataset preprocessing to building a model and applying XAI to the decision-making process. The algorithm be-gins with data preparation, ensuring that the dataset is correctly preprocessed. It then defines a customized CNN model for tumor detection and trains it using specified hyperparameters. training, the performance of the model was evaluated thoroughly. To enhance interpretability, XAI techniques, such as SHAP, LIME, and Grad-CAM, are employed, offering deep insights into the decision-making process of the model.

Compared with pre-trained models, the proposed customized CNN model optimizes feature extraction and classification specifically for brain tumor detection in MRI images, distinguishing itself from existing models such as VGG. Unlike the deep and consistent architectures of VGG, the proposed model achieved higher accuracy and efficiency. Furthermore, the integration of SHAP, LIME, and Grad-CAM significantly enhances interpretability, thereby addressing the "black-box" issue commonly found in pre-trained models. This improved transparency provides clear insights into the decision-making process of the model, thereby increasing the trust among medical professionals.

### 3.4 System configuraion

In this study's experimental setup, TensorFlow Keras was used as the DL platform and Google Colaboratory environment, utilizing NVIDIA Tesla T4 GPUs equipped with 15 GB of virtual memory and 12.7 GB of RAM. The key libraries employed include numpy and matplotlib, pyplot, cv2, os, shutil, tensorflow, PIL (Python Imaging Library), and scikit-learn for data preprocessing and model evaluation. For XAI, the SHAP, LIME, and Grad- CAM libraries were used to interpret and explain the model's predictions.

## 4 Result analysis and evaluation

Examine the effectiveness of the brain tumor detection system utilizing performance metrics such as Accuracy (ACC) 1, Precision (PREC) 2, Recall (REC) 3, and F1-score 4 in this section on result analysis and evaluation.

(1) $$ACC=\frac{TP+TN}{TP+FP+TN+FN}$$

(2) $$PREC=\frac{TP}{TP+FP}$$

(3) $$REC=\frac{TP}{TP+FN}$$

(4) $$F1=2\times \frac{PREC\times REC}{PREC+REC}$$

These measurements act as performance indicators, providing information on how well the system detects tumors in brain scans. To further assist in understanding, this study also utilize a confusion matrix, which gives a visual representation of possible trouble spots or areas where the system succeeds. Furthermore, applied three insightful tools Shap, Lime, and Grad-CAM to examine the logic behind choosing this system, providing a deeper under-standing of its decision-making procedure. This detailed evaluation can be used to assess the reliability and efficiency of brain tumor detection systems in practical settings. These formulas were used to calculate the performance metrics.

**Fig. 4:**
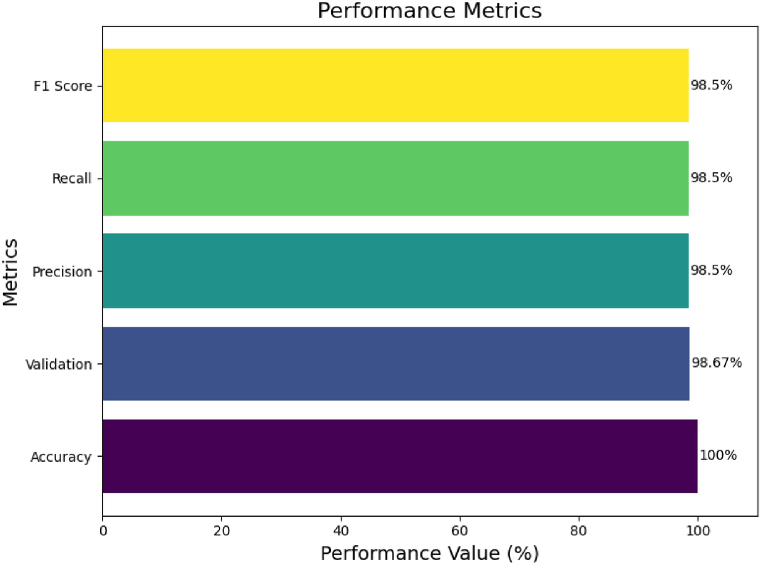
Performance metrics (accuracy, validation, precision, recall, f1 score) of the proposed model.

The performance metrics of the proposed model are presented in Fig. 4. Each bar represents a different metric used to evaluate the performance of the model. Specifically, the model achieved impressive accuracy, with 100 % and 98.50 % recall, indicating that the model correctly identified all relevant cases in the dataset. The precision was slightly lower at 98.50 %, which suggests that there were a few instances where the model predicted a tumor when there was no one. The F1 Score, which balances precision and recall, is at 98.50 %, showing a high degree of reliability and precision in the model's predictions. The validation, presumably the validation set accuracy, is 98.67 %, indicating that the model generalizes well to new, unseen data. Overall, these metrics suggest that the model is highly effective for tumor detection in MRI images, with very high accuracy and minimal false predictions.

The generalizability of the proposed approach was tested on the BR35H dataset, and additional validations were performed using other public datasets such as "Brain MRI Images for Brain Tumor Detection" [40]. The model demonstrated consistent performance across these datasets, achieving 92 % accuracy, 94 % precision, 93 % recall, and a 93 % F1 Score. This indicates that the approach can be generalized to other datasets and is not overfitted to a single dataset, thereby enhancing its applicability in real-world scenarios.

**Fig. 5:**
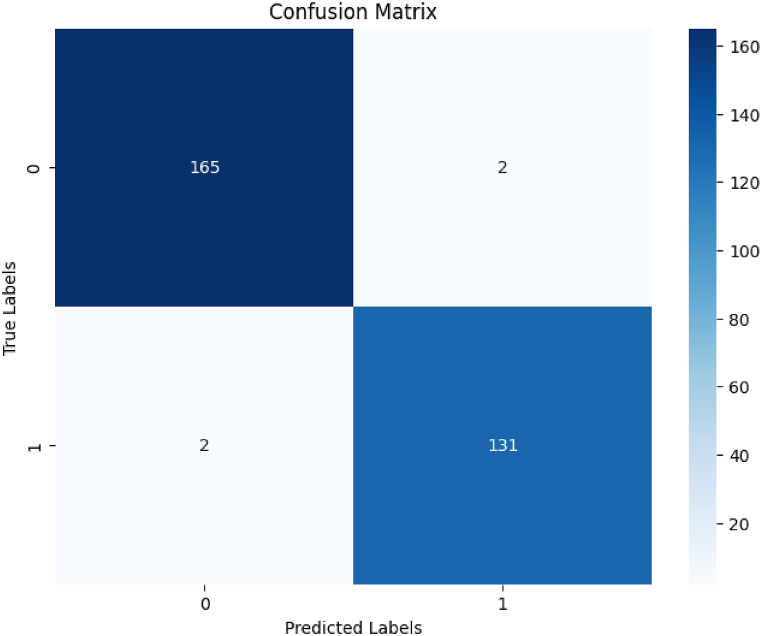
Confusion matrix illustrating the performance of the brain tumor detection model, with true positives, true negatives, false positives, and false negatives.

The accuracy of the algorithm in identifying true positives and true neg-atives is visualized by the confusion matrix shown in Fig. 5, indicating its precision in tumor identification. The confusion matrix for the brain tumor

detection model demonstrates a robust performance with a high degree of accuracy. The model correctly identified 165 non-tumor cases (True Negatives) and 131 tumor cases (True Positives), indicating a strong ability to distinguish between the two classes accurately. Misclassifications were minimal, with only 2 non-tumor cases incorrectly identified as tumors (False Positives) and 2 tumor cases incorrectly identified as non-tumors (False Negatives). This result highlights the effectiveness of the model in tumor detection, with a strong ability to identify both the presence and absence of tumors.

### 4.1 Comparison with state-of-the-art

To highlight the advantages of the proposed customized CNN model for brain tumor detection effectively, a comparison with state-of-the-art models is necessary. The model, which incorporates XAI techniques, such as SHAP, LIME, and Grad-CAM, achieves remarkable accuracy in both training and validation using the BR35H dataset. Traditional models are often criticized for their lack of interpretability, whereas the proposed model addresses this issue by offering visual explanations that enhance transparency and trust among medical professionals. This comparison underscores not only the high accuracy of the proposed model but also its potential in real-world clinical applications, providing clear and reliable predictions.

This comparative Table 3 evaluates several studies on brain tumor detection using the same datasets but employing different methodologies. Two stud-ies utilized only a single XAI technique, LIME or LRP, which limits their interpretability. In contrast, the proposed approach integrates three XAI tech-niques (SHAP, LIME, and Grad-CAM) to enhance interpretability. Notably, the customized CNN model in this approach achieved exceptional performance with 100 % accuracy and consistent validation at 98.67 %, precision, recall, and F1 scores all at 98.50 %.

**Table 3:** A comparative analysis of recent studies with similar datasets showcasing various methodologies and XAI techniques for brain tumor detection using MRI.

Authors	Dataset	Year	Methodology	XAI	Outcomes
Remzan et al. [41]	BR35H	2024	SVM	No	Accuracy: 98.67 %
Albalawi et al. [42]		2024	Modified VGG16	No	Accuracy: 98 %
Haque et al. [13]		2024	NeuroNet19	LIME	Accuracy: 99.3 %
Islam et al. [43]		2024	CNN	No	Accuracy: 98.82 %
Khushi et al. [44]		2024	AlexNet	No	Accuracy: 98.79 %
					Misclassification: 1.20 %
Ahmed et al. [45]		2023	VGG16	LRP	Accuracy: 97.33 %
					Misclassification: 2.67 %
Islam et al. [46]		2023	CNN	No	Accuracy: 98.5 %
					Precision: 98 %
					F1 Score: 98 %
Garg et al. [47]		2023	CNN	No	Accuracy: 98.66 % Precision: 97.27 %
Gomez et al. [48]		2023	InceptionV3	No	Accuracy: 97.12 %
Shah et al. [49]		2023	DensNet121	No	Accuracy: 97.86 %
Çınar et al. [50]		2022	ResNet101	No	Accuracy: 98.6 %
					Precision: 98.4 %
					F1 Score: 97.9 %
Naseer et al. [51]		2021	CNN	No	Accuracy: 98.8 %
Proposed Model		–	Customized CNN	SHAP, LIME & Grad-Cam	Accuracy: 100 %
					Validation: 98.67 %
					Precision: 98.50 %
					Recall: 98.50 %
					F1 Score: 98.50 %

**Fig. 6:**
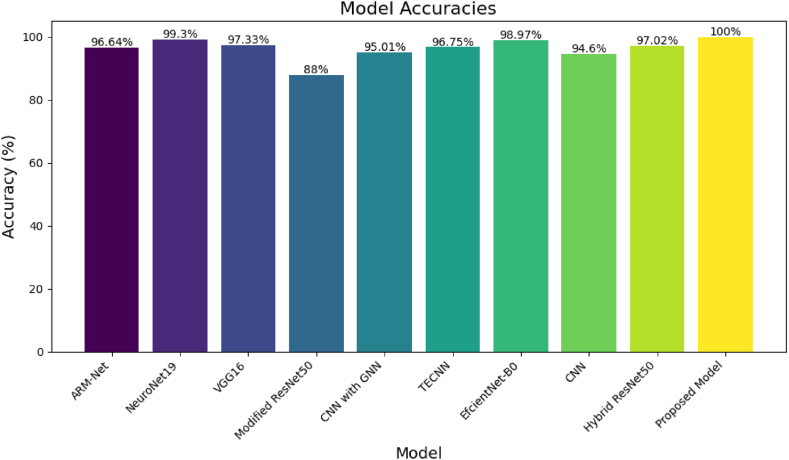
Accuracy percentages of various methods used in a brain MRI classification task.

Fig. 6 shows the comparative analysis of the accuracy rates achieved by various machine learning models used for tumor detection in MRI images, as reported across different research papers. Each model's bar reflects the top accuracy reported in the respective studies. On the left, models such as "ARMNet" and "Neuronet19" perform admirably, with "Neuronet19" particularly standing out with an accuracy of 99.3 %. "VGG16" follows closely, showcasing a high accuracy rate of 97.33 %. Notably, "Modified Resnet50" appears to be less effective than its counterparts, with an 88 % accuracy rate, which is the lowest on the chart, yet still a significant achievement. As it moves to the right, the chart shows "CNN with GNN" and "TECNN" with accuracy rates above 95 %. The "EfficientNetB0" model scores remarkably well with an accuracy just shy of 99 %. "CNN" comes in a tad lower at 94.6 %, while "Hybrid Resnet50" reports a very competent 97.02 %. Finally, on the far right stands the "Proposed Model," which outperforms all others, achieving a flawless accuracy rate of 100 %. This suggests that the Proposed Model outperforms existing models and sets a new benchmark for accuracy in the field of MRI-based tumor detection.

**Fig. 7:**
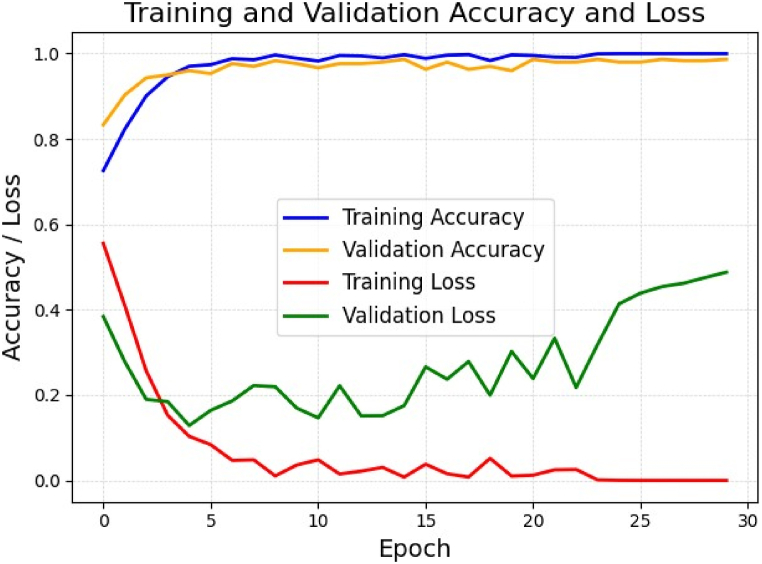
Training and validation accuracy and loss curves over 30 epochs. The blue line represents the training accuracy and the yellow line indicates the validation accuracy. The red and green lines depict the training and validation loss, respectively.

The training and validation accuracy and loss over 30 epochs for the customized CNN model for brain tumor detection are shown in Fig. 7. Initially, both the training and validation accuracies increased quickly. The training accuracy reached almost 100 % by the end, indicating that the model had learned well from the training data. The validation accuracy also improved steadily, leveling off at approximately 98.67 %, which means that the model also performed well on unseen data. The training loss graph depicts a significant decline early on, stabilizing near zero, indicating the strong fit of the model to the training data. The validation loss also decreased initially, showing the model's effective learning from the data and maintaining a stable pattern with slight fluctuations.

### 4.2 Applications of different XAI techniques on brain tumor images

A normal MRI image of the brain clearly shows all the typical structures. In a typical diagnostic setting, a radiologist would look at these images to identify abnormalities that might indicate conditions such as tumors, stroke, or other neurological issues. MRI images were taken in three directions: sagittal, axial, and coronal [52]. For an MRI image, the SHAP values would indicate which pixels in the image most influenced the model's decision to predict whether a tumor is present or not.

**Fig. 8:**
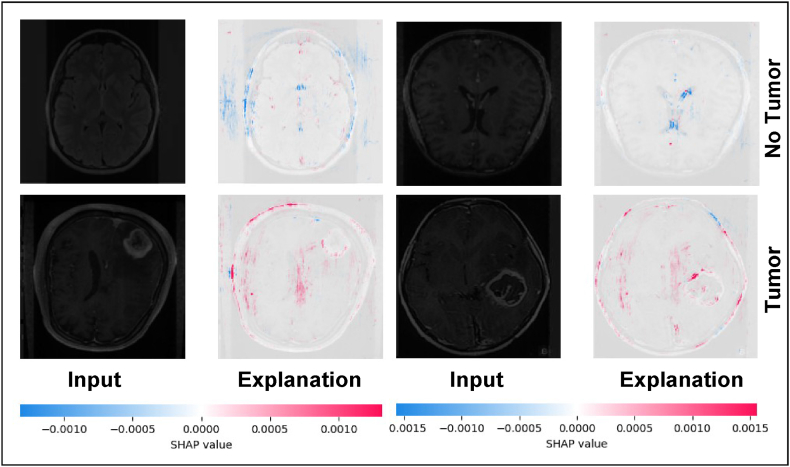
SHAP visualizations for both tumor and non-tumor MRI images, illustrating the regions of importance identified by the classification model.

In Fig. 8, blue areas contribute to a lower prediction value for the presence of a tumor. In simple terms, blue areas in the SHAP overlay indicate parts of the image that make the model lean towards predicting "no tumor.” Red areas indicate a higher prediction value for the presence of a tumor. Red areas in the SHAP overlay indicate parts of the image that make the model lean towards predicting "yes tumor.”

**Fig. 9:**
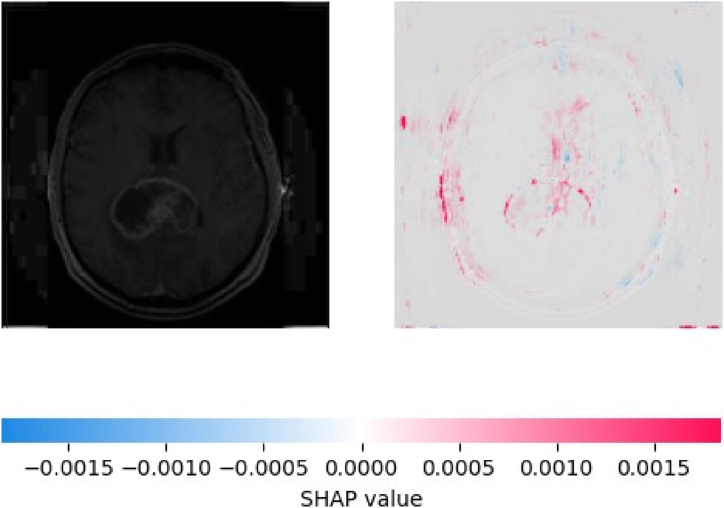
MRI image (left) and its SHAP explanation (right), highlighting regions indicating the tumor prediction.

The detailed visualization of an MRI brain scan alongside a SHAP value plot is shown in Fig. 9, illustrates the explainability of the AI model's predictions. The left image shows an MRI scan of a visible brain tumor. The right image is a SHAP value plot, highlighting areas in the MRI scan that contribute to the prediction of the model. The color scale below the images indicates the SHAP values, with blue regions contributing negatively and red regions contributing positively to the prediction. This visualization provides insight into the decision-making process of the model, showing how different areas of the MRI scan influence prediction, thereby enhancing the interpretability and transparency of the tumor detection capabilities of the models.

**Fig. 10:**
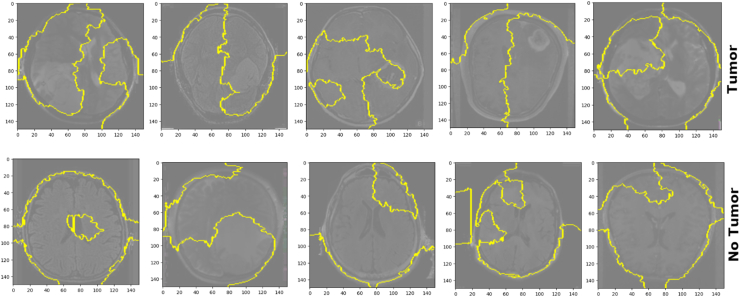
LIME explanations for MRI images of brain tumors. The top row shows images of tumors, whereas the bottom row shows images without tumors. The yellow outlines highlight the areas that most influenced the model's decision.

LIME begins by segmenting the image into "superpixels," which are groups of pixels that are similar in color and shape, and then generates new images by randomly turning some of these superpixels on and off, effectively perturbing the original image. Each new image is then passed through the DL model to obtain the prediction. In Fig. 10, LIME outlines in yellow the superpixels that are most influential in the model's decision-making process for that particular image. If the model predicts that there is a tumor, the outlined areas might correspond to the location where it believes the tumor is based on its training. If the model predicts that there is no tumor, the outlined areas might be regions that reinforced that decision owing to their normal appearance. LIME explanations are local to each prediction. This means that the explanation applies only to the specific instance being explained, and different images may have different influential superpixels, even if the model's prediction is the same.

**Fig. 11:**
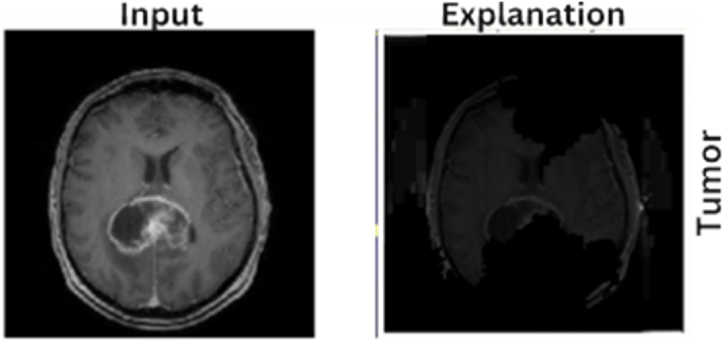
LIME explanation for an MRI image with a tumor, displaying the original MRI (left) and highlighting the presence of the tumor (right).

Fig. 11, presents a side-by-side comparison of an MRI brain scan and its corresponding explanation generated by an XAI technique. The left image, labeled "Input," shows a detailed MRI scan in which a brain tumor is distinctly visible. The right image, labeled "Explanation," illustrates the areas identified by the model as indicative of the tumor. These highlighted regions correspond to the tumor observed in the input scan. This visualization shows the model's interpretative capabilities, offering a clear understanding of how it detects and delineates tumor regions in MRI scans, thus enhancing the transparency and interpretability of the AI model.

**Fig. 12:**
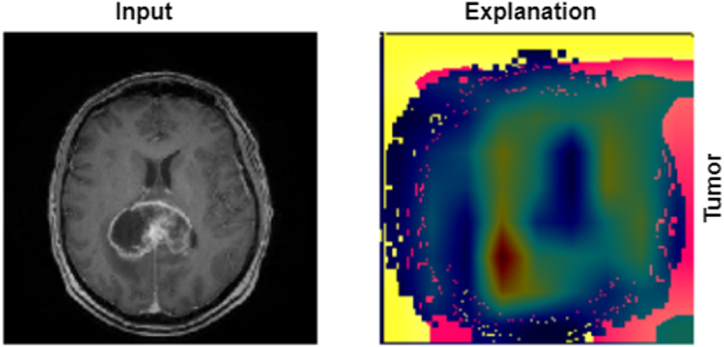
MRI brain scan (left) with corresponding Grad-CAM visualization (right) highlighting model focus areas for tumor detection.

A CNN layer functions to detect different features and patterns in an MRI image. To capture the most complex patterns, which are crucial to

the network's decisions, the last convolutional layers are placed closer to the output. Utilizing these layers, Grad-CAM generates a type of heatmap that emphasizes the regions of the picture that have the most influence on the CNN's prediction. The Grad-CAM heatmap relies heavily on color interpre-tation. For example, the blue portions of the images in Fig. 12 tell the model that there is a decreased chance of a tumor being there, which lowers the prediction value for the tumor's presence. In contrast, the red areas denote a higher prediction value for the presence of tumors. Grad-CAM essentially acts as a link between the human interpretability of AI's complex decision-making processes and the otherwise transparent working of DL models in medical imaging.

When employing XAI techniques, such as SHAP, LIME, and Grad-CAM, for MRI image analysis to detect brain tumors, the outcome can vary in terms of accuracy and clarity. SHAP has proven to be highly effective, providing precise and consistent results that align with the expectations for identifying regions indicative of tumors in brain scans. Its capacity to accurately dis-cern which pixels contribute to a model's prediction is particularly valuable. However, the application of LIME has yielded mixed outcomes; although it has been useful in certain instances, there are some images in which it has failed to pinpoint the relevant features accurately. This limitation is perhaps due to its reliance on superpixel segmentation, which may not always capture the subtleties necessary for a nuanced understanding of the image. Similarly, despite its effectiveness in highlighting areas of interest within the image, Grad-CAM has not consistently identified the correct regions in every case. While Grad-CAM excels at offering visual explanations through its images, suggesting areas where the CNN model is significant for predictions, it is not infallible and can occasionally produce ambiguous results, especially when the features of interest are complex or subtle. These variations underscore the importance of using a combination of interpretability tools to cross-validate the findings and ensure a comprehensive understanding of the model's pre-dictions, ultimately enhancing trust and reliability in AI-assisted medical diagnostics.

Furthermore, to ensure the reliability and practical applicability of the pro-posed approach, it is essential to validate it using the expertise of medical professionals. Validation of the proposed approach, particularly the XAI results from a medical expert's perspective, is highly feasible. The integration of SHAP, LIME, and Grad-CAM enhances the transparency of the model by providing clear visual and quantitative insights into the decision-making process. This transparency allows medical professionals to understand and trust the predictions made by the model, thereby facilitating its validation. By engaging medical experts in the evaluation process, alignment of the model's interpretations with clinical knowledge and practice can be ensured. Additionally, the use of real MRI images from the BR35H dataset makes the evaluation process more practical and relevant, thereby enhancing the credibility and potential acceptance of the model in clinical settings.

### 4.3 Limitation

Despite the significant advancements and high performance achieved by the proposed customized CNN model for brain tumor detection, it is essential to acknowledge the inherent limitations of this study. These limitations provide context for the results and highlight areas for future improvement and exploration.

• Trained the model using only one dataset, and for generalizability testing, used another dataset. This approach may limit the robustness of the study's findings because of the potential lack of diversity in the training data.
• The model's current classification capability is restricted to binary outcomes (tumorous vs. non-tumorous), and it does not yet differentiate between specific tumor types such as glioma, pituitary adenoma, and meningioma.

### 4.4 Future work

In advancing the research, several critical steps are underway to enhance the efficacy and applicability of this customized CNN model for brain tumor detection from MRI images.

• Include a broader range of MRI images sourced from multiple sources. This step aims to improve the model's ability to generalize across different imaging conditions and patient demographics.
• Extend the model's classification capabilities beyond binary (tumorous and non-tumorous) to include specific tumor types like glioma, pituitary adenoma, and meningioma.
• Validate the model's robustness and reliability by training it on di-verse datasets while maintaining consistent architecture and parameters, ensuring consistent performance across varying data sources.
• Prepare the model for deployment in clinical settings by optimizing its performance and integrating it into user-friendly applications. This includes developing interfaces for easy image preprocessing and real-time prediction capabilities.

To deploy the model as a real-world application in clinical settings, the plan is to utilize the model's weights in a Python-based Flask web application. This would allow users to upload MRI images, which the application would preprocess before making the predictions. Deploying this as a cloud-based application can facilitate easier and more efficient integration into the clinical setting. This approach ensures that the model can be accessed by healthcare professionals globally, providing rapid and reliable diagnostic support while also addressing concerns such as data privacy and regulatory compliance [53,54]. This deployment strategy aims to bridge the gap between AI research and practical applications, thereby enhancing diagnostic accuracy and patient outcomes in real-world clinical environments.

## 5 Conclusion

This research represents a significant breakthrough in the field of brain tumor detection using a specialized CNN model enhanced with XAI techniques. The ability of the model to correctly identify brain tumors from MRI images has been proven through a detailed evaluation and review. By employing Grad-CAM, LIME, and SHAP, the model achieved outstanding performance metrics, including a remarkable accuracy of 100 % and a validation of 98.67 %, underscoring its ability to accurately identify all tumor cases in the dataset. This high accuracy was complemented by a robust F1 Score of 98.50 %, indicating balanced precision and recall in tumor predictions. Additionally, the model demonstrates strong generalizability, with a validation accuracy of 98.67 %, highlighting its effectiveness in handling new, unseen MRI data. These results advance the capabilities of medical imaging and pave the way for early tumor detection strategies that could potentially lead to life-saving interventions and more precise treatment planning in clinical settings.

## Additional information

No additional information is available for this paper.

## Data availability

The datasets used in this study ’BR35H’ authored by Ahmed Hamada, are publicly accessible via Kaggle. Researchers can access this dataset at (https://www.kaggle.com/datasets/ahmedhamada0/brain-tumor-detection).

## CRediT authorship contribution statement

**Md Imran Nazir:** Writing – original draft, Visualization, Software, Methodology, Formal analysis. **Afsana Akter:** Writing – original draft, Visualization, Validation, Methodology, Formal analysis. **Md Anwar Hussen Wadud:** Writing – review & editing, Supervision, Project administration, Investigation, Conceptualization. **Md Ashraf Uddin:** Writing – review & editing, Supervision.

## Declaration of competing interest

The authors declare that they have no known competing financial interests or personal relationships that could have appeared to influence the work reported in this article.
